# Reduced number of IFN‐γ producing cells in peripheral blood is a biomarker for patients with renal cell carcinoma

**DOI:** 10.1002/iid3.637

**Published:** 2022-06-06

**Authors:** Kun‐Jin Wu, Kun Yang, Feng‐Ping Zhang, Si‐Nan Liu, Kai‐Bo Yang, Xiao‐Hua Ma, Xing Zhang, Yan‐Fen Ma, Hui Geng, Zheng Wang, Chang Liu, Ting Lin

**Affiliations:** ^1^ Department of Hepatobiliary Surgery The First Affiliated Hospital of Xi'an Jiaotong University Xi'an China; ^2^ Department of Surgical ICU The First Affiliated Hospital of Xi'an Jiaotong University Xi'an China; ^3^ Department of Clinical Laboratory 临床检验科 The First Affiliated Hospital of Xi'an Jiaotong University 西安交通大学第一附属医院 Xi'an China 习安中国; ^4^ Physical Examination Center The First Affiliated Hospital of Xi'an Jiaotong University Xi'an China

**Keywords:** animals, cells, human, molecules, cytokines, T cells, thelper cells (Th1/Th2/Th17)

## Abstract

Renal cell cancer (RCC) is the most lethal of all the common urologic cancers and constitutes 2.2% of all malignancy diagnoses. The incidence of RCC has been steadily increasing in recent decades. The classic risk factors of RCC include smoking, hypertension, obesity, genetics, and genetic mutations. Recent studies also revealed that RCC was an immunogenic tumor and affected by host immune status. Among the pan‐cance, RCC presented with the highest degree of immune infiltration, indicating RCC patients might benefit from immunotherapy. A new immune classification of RCC has been developed by Su et al. based on tumor‐infiltrating lymphocytes to guide clinical practice. However, these studies mainly focus on biomarkers derived from tumor microenvironment (TME), the biomarkers based on peripheral blood samples to RCC have rarely been described. We collected peripheral blood samples from RCC patients and their matched healthy controls and detected the number of IL‐2 and IFN‐γ producing cells by implementing an enzyme‐linked immunospot (ELISPOT) assay. This is the first study to report blood‐based immune biomarkers for RCC using an ELISPOT assay. Our results suggested the frequency of IFN‐γ producing cells but not IL‐2 producing cells was associated with RCC risk. These findings warrant further validation in larger prospective studies.

Renal cell cancer (RCC) is the most lethal of all the common urologic cancers and constitutes 2.2% of all malignancy diagnoses.^1^ The incidence of RCC has been steadily increasing in recent decades. The classic risk factors of RCC include smoking, hypertension, obesity, genetics, and genetic mutations.^2^, ^3^ Recent studies also revealed that RCC was an immunogenic tumor and affected by host immune status.^4^ Among the pan‐caner, RCC presented with the highest degree of immune infiltration, indicating RCC patients might benefit from immunotherapy.^5^ A new immune classification of RCC has been developed by Su et al. based on tumor infiltrating lymphocytes to guide clinical practice.^6^ However, these studies mainly focusing on biomarkers derived from tumor microenvironment (TME), the biomarkers based on peripheral blood samples to RCC have rarely been described.

To find a simple method that could be easily used in clinic, we designed the present case‐control study and explored blood biomarkers reflecting RCC patients' immune response. We collected peripheral blood samples from RCC patients and their matched healthy controls, and detected the number of IL‐2 and IFN‐γ producing cells by implementing an enzyme‐linked immunospot (ELISPOT) assay. ELISPOT assay was a widely used method for detection of the immune response of tuberculosis patients, but it was rarely implemented in tumor patients. Except of the simple and inexpensive advantages, ELISPOT could give a global view of cytokine‐producing cell status which had potential to reflect patients' overall immune status. To our knowledge, this is the first study of functional immune cells as potential biomarkers for RCC risk using ELISPOT assay.

A total of 123 patients with newly diagnosed, histologically confirmed RCC and 60 age‐and gender‐matched healthy controls were recruited in this case‐control study (Table S1). We enumerated the frequencies of cells producing IL‐2 and IFN‐γ in 100,000 PBMCs. The frequency of IFN‐γ producing cells was significantly lower in cases than in controls (*p* = .028); however, there were no significant difference in IL‐2 producing cells (Figure S1). We further analyzed the effect of host characteristics (age, sex, smoking status, hypertension, and BMI) on the frequencies of cells producing IL‐2 and IFN‐γ. In the control group, females had significantly lower frequency of cells producing IFN‐γ than males (*p* = .014). Other key risk factors for RCC, such as age, smoking, hypertension and BMI, did not have significant effect on the frequencies of cells producing IL‐2 and IFN‐γ (Table S2). We used unconditional logistic regression analysis to assess the association between these markers and RCC risk. Either dichotomized at the median or split into tertiles, individuals with low frequency of IFN‐γ producing cells had a significantly increased risk of RCC (*p* = .012 for median and *p* = .003 for tertile; Table 1). There was no association between the frequency of IL‐2 producing cells with RCC risk either in overall or stratified groups (Table 1).

**Table 1:** Association of the frequency of IFN‐γ and IL‐2 producing cells with RCC risk.

Variables	Cases, *n* (%)	Controls, *n* (%)	aOR (95% CI)^a^	*p*
***Frequency of IFN‐γ producing cell in 100,000 PBMCs***				
By median				
>700	42 (34.15)	30 (50.00)	1 (reference)	‐
≤700	81 (65.85)	30 (50.00)	2.30 (1.17–4.52)	**.016**
By tertile				
>980	22 (17.89)	20 (33.33)	1 (reference)	‐
499–980	38 (30.89)	20 (33.33)	1.93 (0.82–4.48)	.129
≤498	63 (51.22)	20 (33.33)	3.50 (1.51–8.08)	**.003**
*p* for trend				**.003**
***Frequency of IL‐2 producing cell in 100,000 PBMCs***				
By median				
>256	56 (45.53)	30 (50.00)	1 (reference)	‐
≤256	67 (54.47)	30 (50.00)	1.11 (0.60–2.08)	.736
By tertile				
>328	41 (33.33)	20 (33.33)	1 (reference)	‐
171–328	41 (33.33)	20 (33.33)	1.06 (0.49–2.29)	.884
≤170	41 (33.33)	20 (33.33)	1.09 (0.50–2.38)	.835
*p* for trend				.835

Abbreviations: 95% CI, 95% confidence interval; aOR, adjusted odds ratio; BMI, body mass index; RCC, renal cell carcinoma.

^a^ OR was adjusted for age, sex, and smoking.

IFN‐γ is a pleiotropic cytokine with multiple biological effects, one of which is a direct cytotoxic effect on tumor cells.^7^ IFN‐γ is known to be produced by NK cells, activated T cells, B cells, and antigen‐presenting cells.^8^, ^9^ However, whether these IFN‐γ producing cells were associated with RCC remains unclear. Our results firstly confirmed that the frequency of IFN‐γ producing cells in peripheral blood samples was significantly lower in RCC patients, indicating that host immune deficiency might be an important clinical characteristic in those patients.

Since cytokines have been associated with RCC risk, we measured a number of cytokines in the supernatant of stimulated PBMCs. Two pro‐cancer cytokines, IL‐6 and IL‐10, were significantly associated with an increased risk of RCC (Table S3). We further assessed the joint effects of the low frequency of IFN‐γ producing cells and these two cytokines on the risk for RCC. Individuals were categorized into four groups by the IFN‐γ producing cells (high or low as dichotomized by the 33rd percentile value in controls) and by cytokine variables (high or low as also dichotomized by the 33rd percentile value in controls). Individuals who had neither high frequency of IFN‐γ producing cells and low IL‐6/IL‐10 levels were used as the reference group. Compared with this group, “low frequency of IFN‐γ producing cells and high IL‐6 level” and “low frequency of IFN‐γ producing cells and high IL‐10 level” groups showed a dramatically increase of RCC risk with ORs of 3.78 (95% CI: 1.25–11.41; *p* = .018) and 4.51 (95% CI: 1.51–13.51; *p* = .007), respectively (Table 2). Our data showed strong‐interaction effects between functional T cells and pro‐cancer cytokines.

**Table 2:** Joint effects of *IFN‐γ* producing cells and IL‐6/IL‐10 levels on RCC risk

Variables	Cases, *n* (%)	Controls, *n* (%)	aOR (95% CI)^a^	*p*
***IFN‐γ*** **producing cells** ***and IL‐6***				
IFN‐γ + cell ^high^/IL‐6 ^low^	19 (65.52)	10 (34.48)	1 (reference)	
IFN‐γ + cell ^high^/IL‐6 ^high^	41 (57.75)	30 (42.25)	0.64 (0.24–1.69)	.367
IFN‐γ + cell ^low^/IL‐6 ^low^	5 (31.25)	11 (68.75)	0.20 (0.05–0.83)	**.027**
IFN‐γ + cell ^low^/IL‐6 ^low^	58 (86.57)	9 (13.43)	3.78 (1.25–11.41)	**.018**
^*p*^ interaction				**<.001**
***IFN‐γ*** **producing cells and IL‐10**				
IFN‐γ + cell ^high^/IL‐10 ^low^	15 (60.00)	10 (40.00)	1 (reference)	
IFN‐γ + cell ^high^/IL‐10 ^high^	45 (60.00)	30 (40.00)	1.13 (0.43–2.97)	.806
IFN‐γ + cell ^low^/IL‐10 ^low^	10 (50.00)	10 (50.00)	0.78 (0.23–2.66)	.697
IFN‐γ + cell ^low^/IL‐10 ^low^	53 (84.13)	10 (15.87)	4.51 (1.51–13.51)	**.007**
^*p*^ interaction				**.030**

Abbreviations: 95% CI, 95% confidence interval; aOR, adjusted odds ratio; BMI, body mass index; RCC, renal cell carcinoma.

^a^ OR was adjusted for age, sex, and smoking.

Experiment evidence revealed that IL‐6 and IL‐10 could inhibit IFN‐γ production during immune response.^10^, ^11^ By joint effect analysis, we also found that the significant interaction exits between the IFN‐γ producing cells and IL‐6/IL‐10. Individuals with low frequency of IFN‐γ producing cells and high IL‐6/IL‐10 levels had an extremely higher risk for RCC, compared with referent group. These results suggest a potential immune biomarkers in peripheral blood for RCC patients.

In conclusion, this is the first study to report blood based immune biomarkers for RCC using ELISPOT assay. Our results suggested the frequency of IFN‐γ producing cells but not IL‐2 producing cells was associated with RCC risk. These findings warrant further validation in larger prospective studies.

## AUTHOR CONTRIBUTIONS

Ting Lin and Chang Liu designed the study, and Kun‐Jin Wu and Kun Yang drafted the first version of the manuscript. Feng‐Ping Zhang, Kun Yang, Xiao‐HuaMa, and Xing Zhang performed experiments and analyzed and interpreted data. Si‐Nan Liu, Yan‐Fen Ma, Hui Geng, and Zheng Wang recruited patients and provided clinical data and patient outcomes. Ting Lin supervised the study. All authors contributed to the final version of the manuscript and approved the submitted version. All authors read and approved the final manuscript.

## CONFLICT OF INTEREST

The authors declare no conflict of interest.

## ETHICS STATEMENT

The study was approved by the Ethics Committee of The First Affiliated Hospital of Xi'an Jiaotong University (Approval number: 2018‐G‐174). All study procedures were performed in accordance with the Declaration of Helsinki. All study subjects provided a written informed consent to blood collection and to the use, analysis, and publication of the generated data.

## Supporting information

Supporting information.

Supporting information.

## Data Availability

The database had been analyzed during the research are available from the corresponding author on reasonable request.
