# Supplementary material for: Reduced number of IFN‐γ producing cells in peripheral blood is a biomarker for patients with renal cell carcinoma
Source: Immun Inflamm Dis. 2022 Jun 6;10(7):e637. doi: 10.1002/iid3.637 (PMC9168551; doi:10.1002/iid3.637)

**Supplementary Figure1. Comparison of the frequency of cells producing IFN-γ (A) and IL-2 (B) in cases (n=123) and controls (n=60). Data are expressed as the frequency of SFCs in total 1x105 PBMCs.**


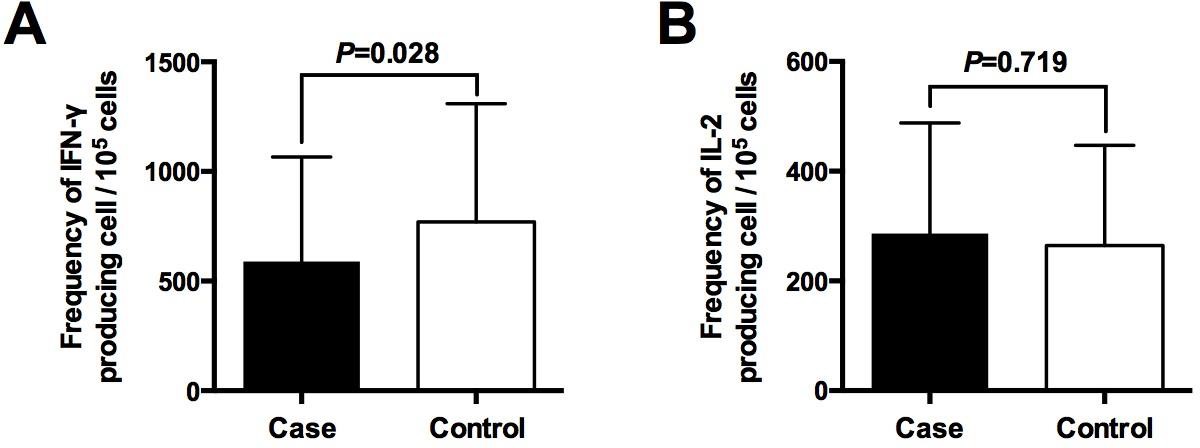

Supplement: Supplementary file 1 — Supporting information. [file IID3-10-e637-s001.docx]
