# Supplementary material for: Reduced number of IFN‐γ producing cells in peripheral blood is a biomarker for patients with renal cell carcinoma
Source: Immun Inflamm Dis. 2022 Jun 6;10(7):e637. doi: 10.1002/iid3.637 (PMC9168551; doi:10.1002/iid3.637)
Supplement: Supplementary file 2 — Supporting information. [file IID3-10-e637-s002.docx]

**Supplementary Table 1. Distributions of selected characteristics between RCC patients and healthy volunteers.**

| **Variables** | | **Cases (n=123)** | **Controls (n=60)** | | | ***P* a** |  |
| --- | --- | --- | --- | --- | --- | --- | --- |
| **Age, mean (SD)** | | 59.18 (8.89) | 59.07 (8.52) | | | 0.935 |  |
| **Sex, *n*(%)**  Male | | 94 (76.42) | 45 (75.00) | | |  |  |
| Female | | 29 (23.58) | 15 (25.00) | | | 0.628 |  |
| **Smoking status, *n* (%)**  Never-smokers | | 57 (46.34) | 33 (55.93) | | |  |  |
| Former-smokers | | 45 (36.59) | 20 (33.90) | | |  |  |
| Current-smokers | | 21 (17.07) | 6 (10.17) | | | 0.349 |  |
| Ever-smokers  Former and current-smokers | | 66 (53.66) | 26 (44.07) | | | 0.226 |  |
| **Mean pack-years of smoking (SD) ^b^** | | 29.83 (26.48) | 29.46 (29.62) | | | 0.628 |  |
| **Hypertension, *n* (%)** ^c^  Yes | | 73 (64.04) | 29 (54.72) | | |  |  |
| No | | 41 (35.96) | 24 (45.28) | | | 0.250 |  |
| **Body mass index, mean (SD)** | | 30.81 (6.53) | | | 30.33 (5.12) | 0.643 | |

Abbreviations: RCC, renal cell carcinoma; SD, standard deviation. ^a^ *P* value was computed by the Pearson χ^2^ test for sex, smoking status and BMI, and was computed by the Student’s *t*-test for age, pack-years and BMI. ^b^ Pack-year only computed in ever- smokers. ^c^ Patients with an “unknown” status were not included.

**Supplementary Table S2. Association of functional T cell number with RCC stratified by host.**

| Variables | IL-2 producing cell number | IFN-γ producing cell number |
| --- | --- | --- |
|  | aOR (95% CI) a P | aOR (95% CI) a P |

| **Age** |  |  |  |  |
| --- | --- | --- | --- | --- |
| ≥ 60 years | 2.22 (0.82-6.01) | 0.117 | 2.51 (0.93-6.78) | 0.069 |
| < 60 years | 0.60 (0.23-1.56) | 0.294 | 2.37 (0.91-6.18) | 0.078 |
| **Hypertension** |  |  |  |  |
| Yes | 1.33 (0.53-3.39) | 0.544 | 2.24 (0.87-5.76) | 0.095 |
| No  **BMI**  ≥30  ＜30 | 1.27 (0.41-3.92)  1.70（0.63-4.60）  1.09（0.38-3.11） | 0.676  0.298  0.868 | 2.27 (0.76-6.81)  1.75（0.64-4.79）  2.28（0.80-6.46） | 0.142  0.280  0.122 |

Abbreviations: RCC, renal cell carcinoma; aOR, adjusted odds ratio; 95% CI, 95% confidence interval; BMI, body mass index. a OR was computed comparing low vs. high level, and was adjusted for age, sex and smoking.

**Supplementary Table 3. Cytokine expression levels in RCC patients and healthy controls**

| **Cytokines ^a^** | **Cases,**  ***n* (%)** | **Controls,**  ***n* (%)** | **aOR**  **(95% CI) ^a^** | ***P* ^b^** |
| --- | --- | --- | --- | --- |
| **IL-4** |  |  |  |  |
| Low | 41 (33.33) | 31 (51.67) | 1 (reference) |  |
| High | 82 (66.67) | 29 (48.33) | 2.39 (1.16-4.91) | **0.018** |
| **IL-5** |  |  |  |  |
| Low | 45 (36.59) | 30 (50.00) | 1 (reference) |  |
| High | 78 (63.41) | 30 (50.00) | 1.40 (0.68-2.88) | 0.362 |
| **IL-6** |  |  |  |  |
| Low | 45 (36.59) | 30 (50.00) | 1 (reference) |  |
| High | 78 (63.41) | 30 (50.00) | 2.03 (0.99-4.14) | 0.052 |
| **IL-8** |  |  |  |  |
| Low | 39 (31.71) | 31 (51.67) | 1 (reference) |  |
| High | 84 (68.29) | 29 (48.33) | 2.86 (1.36-6.02) | **0.005** |
| **IL-10** |  |  |  |  |
| Low | 43 (34.96) | 30 (50.00) | 1 (reference) |  |
| High | 80 (65.04) | 30 (50.00) | 2.49 (1.22-5.09) | **0.012** |
| **IL-12p70** |  |  |  |  |
| Low | 50 (40.65) | 30 (50.00) | 1 (reference) |  |
| High | 73 (59.35) | 30 (50.00) | 1.35 (0.65-2.80) | 0.414 |
| **IL-13** |  |  |  |  |
| Low | 44 (35.77) | 30 (50.00) | 1 (reference) |  |
| High | 79 (64.23) | 30 (50.00) | 1.65 (0.80-3.43) | 0.177 |
| **IL-17A** |  |  |  |  |
| Low | 39 (31.71) | 30 (50.00) | 1 (reference) |  |
| High | 84 (68.29) | 30 (50.00) | 3.24 (1.54-6.80) | **0.002** |
| **TNF-α** |  |  |  |  |
| Low | 45 (36.59) | 30 (50.00) | 1 (reference) |  |
| High | 78 (63.41) | 30 (50.00) | 2.86 (1.36-6.02) | **0.045** |
| **GM-CSF** |  |  |  |  |
| Low | 52 (42.28) | 30 (50.00) | 1 (reference) |  |
| High | 71 (57.72) | 30 (50.00) | 1.13 (0.56-2.30) | 0.725 |

Abbreviations: RCC, renal cell carcinoma; aOR, odds ratio; 95% CI, 95% confidence interval; BMI, body mass index.

^a^ Cytokine levels were categorized by median values in controls as cutoff points.

^b^ Adjusted for age, sex, smoking status, BMI, hypertension and diabetes.
